# Assessing the Influence of Mutation on GTPase Transition States by Using X‐ray Crystallography, ^19^F NMR, and DFT Approaches

**DOI:** 10.1002/anie.201703074

**Published:** 2017-05-24

**Authors:** Yi Jin, Robert W. Molt, Erika Pellegrini, Matthew J. Cliff, Matthew W. Bowler, Nigel G. J. Richards, G. Michael Blackburn, Jonathan P. Waltho

**Affiliations:** ^1^ Department of Molecular Biology and Biotechnology Krebs Institute University of Sheffield Sheffield S10 2TN UK; ^2^ School of Chemistry Cardiff University Cardiff CF10 3AT UK; ^3^ Department of Biochemistry and Molecular Biology Indiana University School of Medicine Indianapolis IN 46202 USA; ^4^ ENSCO, Inc. Melbourne FL 32940 USA; ^5^ Structural Biology Group ESRF-The European Synchrotron, CS40220 38043 Grenoble, Cedex 9 France; ^6^ Manchester Institute of Biotechnology Manchester M1 7DN UK; ^7^ European Molecular Biology Laboratory, Grenoble Outstation CS90181 38042 Grenoble, Cedex 9 France

**Keywords:** ^19^F NMR, DFT calculations, enzyme mechanisms, GTPases, RhoA/RhoGAP

## Abstract

We report X‐ray crystallographic and ^19^F NMR studies of the G‐protein RhoA complexed with MgF_3_^−^, GDP, and RhoGAP, which has the mutation Arg85′Ala. When combined with DFT calculations, these data permit the identification of changes in transition state (TS) properties. The X‐ray data show how Tyr34 maintains solvent exclusion and the core H‐bond network in the active site by relocating to replace the missing Arg85′ sidechain. The ^19^F NMR data show deshielding effects that indicate the main function of Arg85′ is electronic polarization of the transferring phosphoryl group, primarily mediated by H‐bonding to O^3G^ and thence to P^G^. DFT calculations identify electron‐density redistribution and pinpoint why the TS for guanosine 5′‐triphosphate (GTP) hydrolysis is higher in energy when RhoA is complexed with RhoGAP_Arg85′Ala_ relative to wild‐type (WT) RhoGAP. This study demonstrates that ^19^F NMR measurements, in combination with X‐ray crystallography and DFT calculations, can reliably dissect the response of small GTPases to site‐specific modifications.

The combination of X‐ray crystallography, ^19^F NMR spectroscopy and DFT calculations has proved powerful in elucidating the nature of transition states in enzyme‐catalyzed phosphoryl group (PO_3_^−^) transfer reactions.^1^ Importantly, our group recently showed that ^19^F NMR chemical shifts measured for metal fluoride transition‐state analogue (TSA) complexes provide information on the distribution of electron density in the transition state (TS) for PO_3_^−^ transfer.1h, 2 Whether such measurements can also be used to determine changes in TS properties following site‐specific mutations, and hence the functional roles of catalytically important residues, has yet to be established. We investigated this possibility using an archetypal mutation related to the GTPase activity of RhoA, an oncogenic small G‐protein (GTP=guanosine 5′‐triphosphate).

The turnover number of GTP hydrolysis by RhoA is accelerated 3.9×10^4^ fold on binding the GTPase‐activating protein RhoGAP.^3^ The molecular basis of this acceleration has been investigated in structural studies on wild‐type (WT) RhoA/RhoGAP TSA complexes^4^ that conjugate GDP with square planar AlF_4_^−^ or trigonal MgF_3_^−^ surrogates for PO_3_^−^ in the “true” TS. These studies show that the sidechain of a highly conserved arginine (Arg85′ in RhoGAP), termed the “arginine finger”, is inserted into the active site of RhoA in the RhoA/RhoGAP/GTP complex. Four functions have been proposed for the arginine finger in catalysis.^5^ First, its sidechain excludes several water molecules from the active site.^6^ Second, the arginine finger stabilizes an eclipsed orientation of the α‐ and β‐phosphoryl oxygen atoms, adding to the existing strained eclipsing of the β‐ and γ‐phosphoryl oxygens.^7^ Third, the guanidinium moiety facilitates hydrolysis by donating two hydrogen bonds (H‐bonds) to GTP (α‐ and γ‐phosphoryl oxygens).^5^ Fourth, its backbone carbonyl group accepts an H‐bond from the sidechain of a conserved glutamine (Gln63 in RhoA), thereby orientating that carboxamide moiety for catalytic function.^8^ The latter function was expanded recently by the identification of a network of 20 H‐bonds that enables the Gln63 sidechain and the Thr37 carbonyl to act as H‐bond acceptors that orientate an isolated water in the TS for PO_3_^−^ transfer.1h By precluding formation of a H‐bond between this water and the anionic phosphoryl group, this network facilitates shared electron density to stabilize the incipient nucleophile and phosphorus atom.

We now report the integrated use of X‐ray crystallography, NMR, and DFT methods to determine changes in TS properties following site‐directed mutagenesis of Arg85′ to Ala in RhoGAP, and hence to dissect the molecular interactions underlying the deceleration of GTP hydrolysis in the RhoA/RhoGAP complex when the arginine finger is deleted. The X‐ray structure of a TSA complex containing GDP, MgF_3_^−^, and the Arg85′Ala mutant RhoGAP (Arg85′Ala complex) was determined to 2.4 Å using molecular replacement (PDB ID: 5m6x; Figure 1 and Table S1 in the Supporting Information). Difference Fourier maps show clear peaks for the GDP and MgF_3_^−^ ligands, which superimpose well with these ligands in the WT complex (PDB ID: 1ow3; Figure 1). The major difference in the Arg85′Ala complex compared with WT is the conformation of a “Switch I” region close to fluorine atom F^3^ in MgF_3_^−^, which shows a perturbation of the E_32_VYVP_36_ segment. As a result, the aromatic ring of Tyr34 fills space vacated by the deleted Arg85′ sidechain, with the OH hydrogen forming a short H‐bond to F^3^. The structure of the RhoA/RhoGAP_Arg85′Ala_/GDP/AlF_4_^−^ TSA complex was also determined (to 2.2 Å; PDB ID: 5m7o), and showed the same perturbation of Switch I (Figure S1 and Table S1 in the Supporting Information). Switch I is at the interface between RhoA and RhoGAP, and remote from the packing surface with neighboring molecules in the unit cell, thus showing that this perturbed conformation is not a crystallographic artefact. Indeed, this conformation is similar to that reported in the Cdc42/Cdc42GAP_Arg305′Ala_/GDP/AlF_3_ TSA complex (PDB ID: 2ngr).^9^

**Figure 1:**
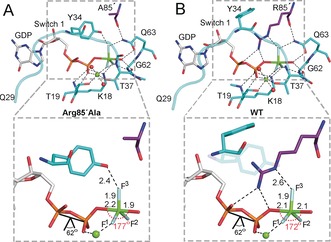
Catalytic core of the MgF_3_^−^ transition‐state analogue for (A) RhoA in complex with GDP and RhoGAP_Arg85′Ala_ (PDB: 5m6x) and (B) the equivalent WT complex (PDB: 1ow3), illustrating the H‐bonding from Tyr34 or Arg85′ to MgF_3_^−^ and GDP (C gray (GDP), cyan (RhoA), or purple (RhoGAP); Mg green; F pale blue; O red; N blue; P orange).

The two Arg85′Ala TSA complexes of RhoA were examined by ^19^F NMR, given the ability of ^19^F chemical shifts to report indirectly on the electronic environment for PO_3_^−^ groups in TSs.1h A single resonance was observed for the AlF_4_^−^ complex (*δ*_average_=−143.6 ppm, Figure S2) due to fast exchange of the four fluorines between the three binding sites for the oxygens of PO_3_^−^.1b,1d,1g Compared with WT, the small upfield chemical shift change (1.6 ppm) and marginal fall in average solvent‐induced isotope shift (SIIS) value (0.2 ppm) for the Arg85′Ala complex indicate that loss of the arginine finger leads to a reduction in the overall tightness of coordination of AlF_4_^−^ (Figure S2).

The MgF_3_^−^ complex exhibits three well‐resolved ^19^F resonances (Figure 2). The assignment of F^1^ is uncomplicated: it has the most upfield resonance, the smallest SIIS value (due to coordination only with the catalytic Mg^2+^ and the backbone NH of Thr 37; Figure 1), and a similar chemical shift to the cognate fluorine in the WT MgF_3_^−^ complex. The similar chemical shifts and SIIS values of the two downfield ^19^F resonances made these assignments more complicated. QM calculations on a geometry‐optimized active site model composed of 95 heavy atoms (190 total atoms) (Figure S3) were therefore used to obtain a preliminary assignment. After optimization, the H‐bond distance from Tyr34‐OH to F^3^ in the model relaxed from 2.4 Å in PDB: 5m6x to 2.58 Å, and ^19^F NMR chemical shifts were calculated (F^1^ −169.8 ppm, F^2^ −148.4 ppm, and F^3^ −160.7 ppm) using standard methods (see the Supporting Information).

**Figure 2:**
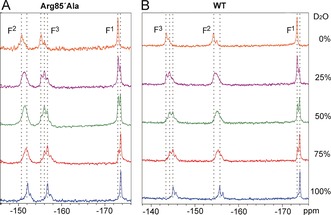
^19^F NMR spectra of RhoA/GDP/MgF_3_^−^ complexes containing RhoGAP_Arg85′Ala_ (A) and WT RhoGAP (B) in aqueous buffer ranging from 0–100 % D_2_O. The spectra are pH‐independent in the range 5–8.5. Chemical shifts (in H_2_O) and SIIS values in the Arg85′Ala complex are −150.8 and 1.2 ppm (F^2^), −155.1 and 1.4 ppm (F^3^), and −173.0 and 0.6 ppm (F^1^). The resonance of F^2^ is broad in mixed H_2_O/D_2_O solutions owing to complex H‐bonding with Lys18 NH_3_^+^.

A partial deuteration strategy experimentally confirmed the DFT‐based assignment of both downfield ^19^F signals. Thus, SIIS values for both WT and Arg85′Ala MgF_3_^−^ complexes were measured in mixed D_2_O/H_2_O solutions (Figure 2 and the Supporting Information). In these spectra, the ^19^F resonance of F^2^, H‐bonded to the Lys 18 ammonium ion, is differentially shifted by HHH, HHD, HDD, and DDD congeners, leading to an unresolved peak in 25–75 % D_2_O/H_2_O solutions. In the WT complex, the middle resonance is unresolved (Figure 2), thus confirming its correct assignment as F^2^.1h In the Arg85′Ala complex, the most downfield resonance is unresolved, thus establishing its assignment as F^2^_._ Hence, F^3^ is the middle resonance in the Arg85′Ala complex (Figure 2), and so removal of the arginine finger has caused the resonance of F^3^ to move markedly upfield (by 11.7 ppm), while the resonance of F^2^ moves downfield by 3.5 ppm. The SIIS values for both F^2^ and F^3^ marginally decrease (by 0.2 ppm) compared to WT, thus implying that MgF_3_^−^ is coordinated less tightly in the mutant complex than in the WT complex.

These ^19^F NMR data indicate that removal of the arginine finger gives rise to a localized electron‐density redistribution within the TSA complex.^10^ We therefore sought to analyze the extent of re‐distribution in the equivalent PO_3_^−^ TS, and thereby assess how the mutation modifies the TS structure. This was accomplished using an optimized computational model of the TS in the RhoA/RhoGAP_Arg85′Ala_/GTP complex (Figure 3), built by replacing the MgF_3_^−^ core in PDB: 5m6x with a trigonal planar PO_3_^−^ moiety (Figure S4). One unique vibrational mode characterizing the motion of P^G^ along the reaction coordinate confirmed that this structure corresponded to a TS (Movie S1). The principal features of the computed TS are “in‐line” trigonal bipyramidal geometry (178.2°), a 4.29 Å apical separation of donor and acceptor oxygens, and a relatively short H‐bond between Tyr34‐OH and O^3G^ (O⋅⋅⋅O, 2.71 Å; Figure 3 and Table S2). For comparison, the values are 177°, 4.1 Å, and 2.4 Å in PDB: 5m6x and 172°, 3.9 Å and 2.6 Å in PDB: 5m7o (Figure 1). The computed TS is unsymmetrical with a P^G^−O^w3^ (nucleophilic oxygen in the attacking water, w3) distance of 1.99 Å, and a P^G^−O^3B^ (leaving group oxygen of GDP) bond length of 2.30 Å (the values computed for these bond lengths in the WT complex are 2.03 Å and 2.19 Å, respectively). A similar non‐symmetry is observed in the TSA complex for MgF_3_^−^ in PDB: 5m6x (Mg−O^w3^=1.9 Å, Mg−O^3B^=2.2 Å). Electron‐density surface maps provide interesting insight into the computational WT and Arg85′Ala transition states. Thus, when contoured at 0.304 e^−^ Å^−3^, the surface density between O^3B^, P^G^ and O^w3^ is continuous in the WT TS whereas it is discontinuous between O^3B^ and P^G^ in the Arg85′Ala TS (Figure 4).

**Figure 3:**
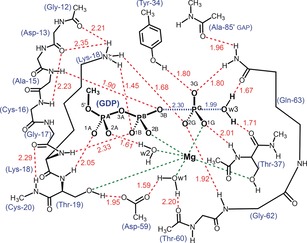
QM‐derived TS model for GTP hydrolysis by RhoA/RhoGAP_Arg85′Ala_ showing the 20 H‐bond network for the catalytic region (red dashes) with the coordination of P^G^ (blue dashes) and Mg (green dashes). Amino acid residues from RhoA and RhoGAP_Arg85′Ala_ that contribute H‐bonds to the network are numbered (H‐bonds H⋅⋅⋅O given in Å).

**Figure 4:**
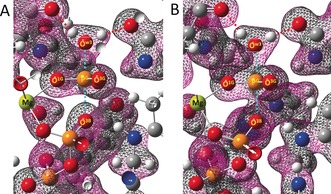
Equivalent sections of the computed electron‐density surface for the catalytic core of Arg85′Ala (A) and of WT (B) transition states for RhoA/RhoGAP‐catalyzed hydrolysis of GTP [contoured at 0.304 e^−^ Å^−3^, (0.045 e^−^ *a*_0_^−3^)]. The reaction coordinate (cyan dashes) is vertical, with O^w3^ top, P^G^ centered, and O^3B^ below. The catalytic magnesium is lower left. Tyr34 is at lower rear of (A) and Arg85′ is at lower rear of (B). O^w3^ donates H‐bonds to the carboxamide C=O of Gln63 (upper right) and to the amide C=O of Thr37 (upper left, red dashed lines). Some frontal atoms and electron‐density mesh removed by “slabbing” for clarity. H white; C gray; O red; N blue; P orange; Mg lime green.

Notwithstanding these differences in electron density, the w3 water hydrogens form regular hydrogen bonds to Thr37 and Gln63 in both computed TSs (Figure 4 and Figure S5). Changes in electron density on the oxygen atoms of the transferring phosphoryl group in TS models for the WT and Arg85′Ala complexes are also evident from calculated ^17^O NMR chemical shifts (Table S3). These suggest that there is a corresponding increase in electron density only on O^3G^ in the Arg85′Ala TS, which corresponds to the changes in ^19^F NMR chemical shifts in the TSA complexes. The marked upfield shift of F^3^ on changing one of its H‐bond partners from Arg85′ to Tyr34 therefore results from significant deshielding by Arg85′ in the WT TSA complex.

Based on the computed bond lengths and ^17^O NMR chemical shift values, we find that the computed TS in the Arg85′Ala complex is later than that calculated for the WT complex.1h This implies that removal of the arginine side chain raises the activation energy for the nucleophilic attack of water on GTP. It is significant that exclusion of water from the TS complex is achieved in the absence of Arg85′. The staggered conformation of the α‐ and β‐phosphoryl oxygens of GTP in the mutant complex is close to that found in the earlier TS computed for WT enzyme, thus leaving no support for the claimed role of the arginine finger in stabilizing a strained, eclipsed conformation for these phosphoryl groups.1h, 7 The reduction in electron density between the migrating P^G^ and O^3B^ in the TS model in the mutant complex relative to WT (Figure 4) indicates that the loss of the H‐bond from Arg85′‐N^η^ to O^3B^ in the Arg85′Ala complex (Figure 1) is of secondary importance. O^3B^ of GDP (p*K*_a3_ is ≈7.2)^11^ is still fully competent to act as a leaving group for nucleophilic attack on P^G^ in the absence of a H‐bond from Arg85′. Moreover, replacing three H‐bonds from Arg85′ by one involving Tyr34 maintains the H‐bond network identified in our earlier study of the WT complex.1h As a result, the WT conformation of the Gln63 carboxamide is maintained, thereby orienting the water correctly for nucleophilic attack.4b, 12

We conclude that loss of the positive charge from Arg85′ and the corresponding impact on its H‐bonding to O^3G^ is the major factor in raising the TS energy for phosphoryl group transfer in the Arg85′Ala complex. Our work therefore supports previous QM/MM studies of the cognate mutation in the Ras/RasGAP complex.8f Thus, RhoGAP binding delivers additional catalysis through displacement of Tyr34 by the arginine finger, thereby enabling the guanidinium group to diminish electron density on P^G^ by the formation of a stronger H‐bond to O^3G^.

Overall, this study shows that ^19^F NMR measurements, in combination with DFT calculations and X‐ray crystal structures, offer a direct experimental method for dissecting the responses of a phosphoryl transfer enzyme to site‐directed residue modification, and can reliably identify even relatively small changes in TS properties. It demonstrates the value of using high‐level QM calculations to interpret ^19^F NMR chemical‐shift changes arising from the effect of site‐specific mutations on the complicated electrostatic environments present in enzyme active sites. These methods also deliver a complete 3D map of electron redistribution in the active site resulting from the mutation. The general strategy reported here opens up the possibility of employing ^19^F NMR measurements to assess the impact of naturally occurring, and in many cases disease‐causing, mutations on the ability of GTPases to catalyze phosphoryl transfer.

## Experimental Section

Experimental methods for X‐ray crystallography, ^19^F NMR, and DFT computations were as described previously,1h and full details are given in the Supporting Information. The nomenclature system used here to described oxygen and phosphorus atoms in the structures is as recommended by IUPAC.^13^

## Conflict of interest

The authors declare no conflict of interest.

## Supporting information

As a service to our authors and readers, this journal provides supporting information supplied by the authors. Such materials are peer reviewed and may be re‐organized for online delivery, but are not copy‐edited or typeset. Technical support issues arising from supporting information (other than missing files) should be addressed to the authors.

Supplementary

Supplementary
