# Supplementary material for: Assessing the Influence of Mutation on GTPase Transition States by Using X‐ray Crystallography, 19F NMR, and DFT Approaches
Source: Angew Chem Int Ed Engl. 2017 May 24;56(33):9732–5. doi: 10.1002/anie.201703074 (PMC5575484; doi:10.1002/anie.201703074)

Angewandte Chemie
Eine Zeitschrift der Gesellschaft Deutscher Chemiker

Supporting Information

Assessing the Influence of Mutation on GTPase Transition States by using X-ray Crystallography, ^{19}F NMR, and DFT Approaches

Yi Jin+, Robert W. Molt, Jr.+, Erika Pellegrini+, Matthew J. Cliff, Matthew W. Bowler, Nigel G. J. Richards, G. Michael Blackburn,* and Jonathan P. Waltho**

anie_201703074_sm_miscellaneous_information.pdf
anie_201703074_sm_R85A_v4.mov

Methods:

Site-directed Mutagenesis

Plasmids expressing N-terminal GST-tagged RhoGAP (fragment 198–439) and N-terminal GST-tagged GST-$RhoA_{F25N}$ were generously given by Dr. K. Rittinger (MRC National Institute for Medical Research, London). The primers for site-directed mutagenesis to make Arg85'Ala mutant of RhoGAP had the sequence shown below:

Forward primer	5'-GAGGGCATCTTCGCGAGGTCGGCC-3'
Reverse primer	5'-GGCCGACCTCGCGAAGATGCCCTC-3'

Tm= 71.3°C, GC%= 70.8%

DNA sequences $p29RhoGAP_{Arg85'Ala}$ mutant was confirmed by DNA sequencing (Source Bioscience-geneservice).

Gene Expression and Protein Purification Both genes for RhoA and $RhoGAP_{Arg85'Ala}$ were expressed in *E. coli.* BL21-Rosetta-pLysS or Rosetta 2 strains in LB media. Expression was induced with 1 mM IPTG for 6 h at 30 °C or overnight at 20 °C, at 250 rpm. Both RhoA and $RhoGAP_{Arg85'Ala}$ were purified following the same protocol. After cells were lysed by sonication in Lysis Buffer (50 mM Tris, pH 7.6, 50 mM NaCl, 5 mM $MgCl_2$, 1 mM DTT), the clear lysate was loaded on glutathione agarose column and incubated at 4 °C on a rotating wheel for 1 h before being washed with thrombin digestion buffer (50 mM Tris, pH 8.0, 150 mM NaCl, 5 mM $MgCl_2$, 1 mM DTT). The on-column thrombin digestion was carried out on beads overnight at 4 °C on a rotating wheel and the flow-through containing impure $RhoGAP_{Arg85'Ala}$ or RhoA was collected. The protein was further purified on a pre-equilibrated S75 Superdex Gel filtration column (50 mM Tris, pH 8.0, NaCl mM 150, 5 mM $MgCl_2$, 1mM DTT. RhoA and $RhoGAP_{Arg85'Ala}$ used for crystallization was additionally purified by size exclusion column in a buffer of 50 mM Bis-Tris (pH 6), 150 mM NaCl, 5 mM $MgCl_2$, and 1 mM DTT. Protein was then concentrated and immediately set up for crystallization experiments.

Crystallization For $RhoA/GAP_{Arg85'Ala}$-GDP-MgF_3^- and $RhoA/GAP_{Arg85'Ala}$-GDP-AlF_4^- TSA complexes, RhoA and $RhoGAP_{Arg85'Ala}$ were mixed in equal molar ratio with NaF (20 mM) and aluminum fluoride (2mM) in the case of the aluminum fluoride metal complex. After overnight incubation, the complex was concentrated to 700 µM. Initial crystallization conditions were established by testing several commercial screens at the EMBL High Throughput Crystallization Laboratory (Grenoble, France) using a Cartesian robot. Crystals were eventually obtained by the microbatch method after several generations of seeding in 100 mM Bis/Tris pH 5.8 or pH 6.0, 23-26% PEG 3350. The crystals obtained were prepared for flash cooling by immersion in the mother liquor made up with 25% PEG 400.

Data Collection and Structure Solution Diffraction data from RhoA/GAP$_{Arg85'Ala}$-GDP-MgF_3^- TSA complexes crystals were collected at 100K to 2.2 Å resolution at beamline ID29 at the ESRF, Grenoble.[1] The structure of the complex was solved by molecular replacement using the previous structure RhoA/GAP$_{wt}$-GDP-AlF_4^- TSA complexes (PDB:**1tx4**) as a search model with the bound ligands and water molecules removed.[2] Data were processed with XDS,[3] and further scaled using AIMLESS.[4] Ligands were included after a few refinement cycles. Refinement was carried out alternately using REFMAC5[5] and manual rebuilding with COOT.[6] Diffraction data from RhoA/GAP$_{Arg85'Ala}$-GDP-MgF_3^- TSA complex crystals at 2.4 Å were collected at 100 K at beam line 14.2 at the BESSY Synchrotron, Berlin. The structure was solved in a similar manner to RhoA/GAP$_{Arg85'Ala}$-GDP-MgF_3^-, using **PDB: 1ow3** as a search model without ligands.[7] Models were validated using Molprobity[8] and structure figures were produced with PyMol.[9][3] and further scaled using AIMLESS. Ligands were included after a few refinement cycles. Refinement was carried out alternately using REFMAC5 and manual rebuilding with COOT. Diffraction data from RhoA/GAP$_{Arg85'Ala}$-GDP-MgF_3^- TSA complex crystals at 2.4 Å were collected at 100 K at beam line 14.2 at the BESSY Synchrotron, Berlin. The structure was solved in a similar manner to RhoA/GAP$_{Arg85'Ala}$-GDP-MgF_3^-, using **PDB: 1ow3** as a search model without ligands. Models were validated using Molprobity and structure figures were produced with PyMol.

NMR Measurements The 1D ^{19}F NMR spectra were recorded on a Bruker Avance 500 MHz spectrometer equipped with a 5 mm cryo $^{1}H/^{19}F$ probe. Typically, 1024 scans were acquired over a spectral width of 200 ppm with the carrier frequency set to –140 ppm. Selective ^{19}F irradiation was achieved with a continuous wave at a power level of 42 dB applied over the 1 s recycle delay at the frequency of free F^- peak. All spectra were recorded at 25 °C.
The RhoA/GAP$_{Arg85'Ala}$-GDP-MgF_3^- TSA samples for NMR analysis contained 1.0 mM RhoA (with stoichiometric GDP bound), 1.1 mM RhoGAP$_{R85'A}$, 50 mM Tris, pH 7.4, 150 mM NaCl,10 mM $MgCl_2$, 10 mM NH_4F, and 1 mM deferoxamine. The RhoA/GAP$_{Arg85'Ala}$-GDP-AlF_4^- TSA samples were prepared by adding 2 mM $AlCl_3$, pH 7.0. For SIIS titrations at 0%, 25%, 50%, 75% and 100% D_2O, the protein samples of RhoA/GAP$_{Arg85'Ala}$-GDP-MgF_3^- complex or RhoA/GAP$_{wt}$-GDP-MgF_3^- complex were concentrated in a Millipore micro-vivaspin at 10k MWCO at 4 °C and exchanged into buffer containing 50 mM Tris buffer (pH 7.4), 150 mM NaCl,10 mM $MgCl_2$, 10 mM NH_4F, and 1 mM deferoxamine with the corresponding percentage of D_2O. The experiments in 100% H_2O used a D_2O capillary as deuterium lock.

A partial deuteration strategy experimentally confirmed the DFT-based assignment of both downfield ^{19}F signals. Thus, the number and size of the contributions of each exchangeable H- bond partner to the SIIS values were determined for both WT and Arg85´Ala MgF_3^- complexes by measuring ^{19}F NMR of the complexes at a variety of H_2O:D_2O ratios (Fig. S2). F^1, with a single exchangeable H-bond partner, has one contribution to its SIIS, and no peaks resulting from partial deuteration are observed. F^2 is H-bonded to the Lys18-NH_3^+ moiety and Gly62-NH, giving potentially 4 unequal contributions to the SIIS. However, rotation of the -NH_3 group averages the influence from the deuterons in the HHD and HDD congeners, producing two equally sized, small contributions to the SIIS at intermediate D_2O ratios. The result is a broad peak composed from five unresolved contributions. In the WT complex, the partially deuterated resonances at -155 ppm (Fig. 2) show this lineshape, confirming its assignment as F^2.[1h] The same lineshape is observed for the most downfield resonance in the Arg85´Ala complex, establishing its assignment as F^2. Since F^3 has two exchangeable H-bond partners, the peaks that resolve into a pseudotriplet at 50% D_2O are assigned to this atom. Hence, F^3 is the middle resonance in the Arg85´Ala complex spectrum (Fig 2), and so removal of the arginine finger causes the resonance of F^3 to move markedly upfield (by 11.7 ppm), while the resonance of F^2 moves downfield by 3.5 ppm. The SIIS values for both F^2 and F^3 marginally decrease (by 0.2 ppm) compared to WT, implying that MgF_3^- is coordinated less tightly in the mutant than in the WT TSA complex.

The DFT-based assignment of the ^{19}F signals was confirmed by determining the contributions of each exchangeable H-bond partner to the SIIS values. For WT and Arg85´Ala MgF_3^- complexes ^{19}F NMR spectra were measured at a variety of H_2O:D_2O ratios (Fig. S2). The F^1 resonance shows one contribution to its SIIS, as anticipated. F^2 is H-bonded to the Lys18-NH_3^+ moiety and Gly62-NH, and rotation of the -NH_3 group averages the influence from the deuterons in the HHD and HDD congeners, producing three equal, unresolved SIIS contributions to the F^2 resonance. At intermediate D_2O:H_2O ratios the result is a broad signal resulting from five contributions. For WT, the middle peak (Fig. 2) shows this behavior, confirming its assignment as F^2.[1h] The same behavior is observed for the most downfield peak of the Arg85´Ala complex, endorsing its assignment as F^2. Since F^3 has two H-bond partners, the peaks that form a pseudotriplet at 50% D_2O are assigned to this atom. Hence for Arg85´Ala, F^3 is the middle peak (Fig. 2). Removal of the arginine finger causes the resonance of F^3 to move markedly upfield (11.7 ppm), while that of F^2 moves downfield (3.5 ppm). The SIIS values for both F^2 and F^3 marginally decrease (by 0.2 ppm) compared to WT, implying that MgF_3^- is coordinated less tightly in the mutant than in the WT TSA complex.

Computational Data for Mutant Transition State Our model for the transition state (TS) of the γ-phosphate hydrolysis reaction was obtained using Kohn-Sham Density Functional Theory (KS-DFT). We used the M06-2X functional formulation of KS-DFT.[10] A cc-pVDZ basis set was used to represent single-particle wave-functions[11] for carbon, hydrogen, oxygen, and nitrogen atoms, while the cc-pVTZ basis was used for magnesium and phosphorus atoms.[11] The active site (cluster) model (Figure 3, main text) was constructed so as to maintain all key hydrogen bonding capable of stabilizing the transition state. The initial geometry about the γ-phosphorus atom was obtained by replacing the tbp magnesium by phosphorus and the three fluorines by oxygens in the high-resolution X-ray structure (PDB: **2ngr**). More specifically, we included atoms in residues 12-20B, 34B, 36-38B, and 59-63B (RhoA) and 85-86A from the crystal structure designation. All amino acid hydrogen bonds stabilizing the attacking water, the γ-phosphoryl group, or leaving group were included. Waters bonded to the catalytic magnesium were also retained. Where opportune, we truncated amino acid residues with a methyl group in which the carbon was fixed at the crystallographic coordinates of the cognate atom in the X-ray crystal structure. Initially, the TS search utilized cc-pVDZ for all atoms and an integration grid consisting of 99 radial points and 590 solid-angle points in the Lebedev grid. Upon calculating an initial TS structure in this manner, we increased the quality of the calculation for greater accuracy and to eliminate spurious small imaginary frequencies. We added basis functions in the manner most conducive of better representing the active site. We improved the phosphorus basis set to cc-pVTZ so as to represent its polarizable electron density with more accuracy. We also increased the magnesium basis set given its strong electron polarization. All oxygen atoms in the phosphate chain used aug-cc-pVDZ, aside the nucleophilic water oxygen, gamma oxygens, and O^{3B} which had aug-cc-pVTZ given the increased importance.[12] The final refined integration grid had 130 radial points, 700 solid angle points. The structure was considered optimized when the force on all nuclei fell below 1 μHartree/Bohr. The SCF was considered converged when the density matrix residual was less than 10^{-6}. After decreasing the initial 1.91 Å Mg-F distance to a value of 1.71 Å for the three new P-O bonds, we optimized the geometry of the resulting active site model (175 atoms) to obtain the TS using standard algorithms,[13] as implemented in the Gaussian09 software package.[14] All methyl carbons were fixed at their initial locations, which did not introduce any significant error into the calculation. This procedure gave a converged TS model with a harmonic vibrational

value of 198*i* cm^{-1} corresponding to motion along the reaction coordinate (see movie S1). However, in freezing the Cartesian coordinates associated with the terminating methyl groups, there were a small number of non-relevant imaginary frequencies associated methyl group librations (37*i* cm^{-1}, 27*i* cm^{-1}, 17*i* cm^{-1}, and 11*i* cm^{-1}).

Obtaining the Calculated Active Site Model for RhoA/GAP$_{Arg85'Ala}$-GDP-MgF_3^- complex An active site model for the RhoA/GAP$_{Arg85'Ala}$-GDP-MgF_3^- complex was obtained from the atomic coordinates of the TS model except that the P and O atoms in the γ-phosphoryl group were replaced by Mg and F, respectively. The optimized structure was obtained using a similar computational protocol to that used for the TS model except that standard optimization algorithms were used to find the ground state structure. Given the electronegativity of fluorine, it was necessary to add diffuse functions in the form of an aug-cc-pVTZ basis on the fluorine atoms.

NMR Chemical Shift Calculations NMR shielding tensors for ^{17}O and ^{19}F nuclei in the TS model and the calculated RhoA/GAP$_{Arg85'Ala}$-GDP-MgF_3^- complex active site model, respectively, were computed from the coupled-perturbed Hartree-Fock equation and gauge-invariant atomic orbitals derived from the DFT electron densities using standard algorithms (Table 3).[15]

References:

[1] D. de Sanctis, A. Beteva, H. Caserotto, F. Dobias, J. Gabadinho, T. Giraud, A. Gobbo, M. Guijarro, M. Lentini, B. Lavault, T. Mairs, S. McSweeney, S. Petitdemange, V. Rey-Bakaikoa, J. Surr, P. Theveneau, G. A. Leonard, C. Mueller-Dieckmann, *J. Synchrotron Rad.* **2012**, *19*, 455-461.

[2] K. Rittinger, P. A. Walker, J. F. Eccleston, S. J. Smerdon, S. J. Gamblin, *Nature* **1997**, *389*, 758-762.

[3] W. Kabsch, *Acta Cryst. D Biol. Crystallogr.* **2010**, *66*, 125-132.

[4] P. R. Evans, *Acta Cryst. D Biol. Crystallogr.* **2011**, *67*, 282-292.

[5] G. N. Murshudov, A. A. Vagin, E. J. Dodson, *Acta Cryst. D Biol. Crystallogr.* **1997**, *53*, 240-255.

[6] a) P. Emsley, K. Cowtan, *Acta Cryst. D Biol. Crystallogr.* **2004**, *60*, 2126-2132; b) P. Emsley, B. Lohkamp, W. G. Scott, K. Cowtan, *Acta Cryst. D Biol. Crystallogr.* **2010**, *66*, 486-501.

[7] D. L. Graham, J. F. Eccleston, C. W. Chung, P. N. Lowe, *Biochemistry* **1999**, *38*, 14981-14987.

[8] V. B. Chen, W. B. Arendall, 3rd, J. J. Headd, D. A. Keedy, R. M. Immormino, G. J. Kapral, L. W. Murray, J. S. Richardson, D. C. Richardson, *Acta Cryst. D Biol. Crystallogr.* **2010**, *66*, 12-21.

[9] The PyMOL Molecular Graphics System, Version 1.8 Schrödinger, LLC.

[10] a) Y. Zhao, D. Truhlar, *Theor. Chem. Acc.* **2008**, *120*, 215-241; b) N. Mardirossian, M. Head-Gordon, *J. Chem. Theor. Comput.* **2016**, *12*, 4303-4325.

[11] T. H. Dunning, *J. Chem. Phys.* **1989**, *90*, 1007-1023.

[12] R. A. Kendall, T. H. Dunning, R. J. Harrison, *J. Chem. Phys.* **1992**, *96*, 6796-6806.

[13] X. Li, M. J. Frisch, *J. Chem. Theor. Comput.* **2006**, *2*, 835-839.

[14] a) M. J. Frisch, G. W. Trucks, H. B. Schlegel, G. E. Scuseria, M. A. Robb, J. R. Cheeseman, G. Scalmani, V. Barone, B. Mennucci, G. A. Petersson, H. Nakatsuji, M. Caricato, X. Li, H. P. Hratchian, A. F. Izmaylov, J. Bloino, G. Zheng, J. L. Sonnenberg, M. Hada, M. Ehara, K. Toyota, R. Fukuda, J. Hasegawa, M. Ishida, T. Nakajima, Y. Honda, O. Kitao, H. Nakai, T. Vreven, J. A. Montgomery Jr., J. E. Peralta, F. Ogliaro, M. J. Bearpark, J. Heyd, E. N.

Brothers, K. N. Kudin, V. N. Staroverov, R. Kobayashi, J. Normand, K. Raghavachari, A. P. Rendell, J. C. Burant, S. S. Iyengar, J. Tomasi, M. Cossi, N. Rega, N. J. Millam, M. Klene, J. E. Knox, J. B. Cross, V. Bakken, C. Adamo, J. Jaramillo, R. Gomperts, R. E. Stratmann, O. Yazyev, A. J. Austin, R. Cammi, C. Pomelli, J. W. Ochterski, R. L. Martin, K. Morokuma, V. G. Zakrzewski, G. A. Voth, P. Salvador, J. J. Dannenberg, S. Dapprich, A. D. Daniels, Ö. Farkas, J. B. Foresman, J. V. Ortiz, J. Cioslowski, D. J. Fox, Gaussian, Inc., Wallingford, CT, USA, **2009**; b) R. Dennington, Keith, T., and Millam, J. Semichem Inc., Shawnee Mission, KS, *Semichem Inc., Shawnee Mission, KS* **2009**.

[15] T. Helgaker, P. Jørgensen, *J. Chem. Phys.* **1991**, *95*, 2595-2601.

[16] G. M. Blackburn, Y. Jin, N. G. Richards, J. P. Waltho, *Angew. Chem. Int. Ed.* **2017;** DOI: 10.1002/anie.201606474.

Movie S1. Visualization of the characteristic imaginary vibrational mode of the TS model in which the water nucleophile attacks the P^G of GTP. Images were created in GaussView V5.0[14b] are looped for ease of viewing. The scalar amplitude of the atomic motions along the eigenvector associated with this vibrational mode is defined by a factor A, which is computed according to the following equation:[14b] are looped for ease of viewing. The scalar amplitude of the atomic motions along the eigenvector associated with this vibrational mode is defined by a factor A, which is computed according to the following equation:

$$A = 0.1 \times [2.0 + (S - 2.0) \times (13.0 / 98.0)]$$

where *S* is an integer value that ranges from 2 to 100. Hence, *A* ranges from 0.2-1.5 Å.

Table S1. Data collection and refinement statistics.

	RhoA/RhoGAP$_{Arg85'Ala}$-GDP-AlF$_4^-$	RhoA/RhoGAP$_{Arg85'Ala}$-GDP-MgF$_3^-$
**PDB**	**5m70**	**5m6x**
**Data collection**		
Space group	$P2_1$	$P2_1$
Cell dimensions		
a, b, c (Å)	72.56, 66.06,76.76	73.74, 66.69, 76.89
α, β, γ (°)	90.00, 96.07, 90.00	90.00, 95.41, 90.00
Resolution (Å)	48.72 (2.2)*	40.29 (2.4)
R_{sym} or R_{merge}	0.077 (0.502)	0.179 (0.661)
$I/\sigma I$	7.9 (1.8)	5.3 (1.9)
Completeness (%)	98.5 (99.0)	84.3 (87.4)
Redundancy	3.0 (3.0)	3.0 (2.8)
**Refinement**		
Resolution (Å)	2.2	2.4
No. reflections	34416	23276
$R_{work/}$ R_{free}	23.81/27.65	22.43/27.20
No. atoms	5933	6107
Protein	5752	5846
GDP/Mg^{2+} /AlF_4^-/MgF_3^-	56/2/10	56/2/8
Water	113	183
B-factors		
Protein	42.19 (chain A)	22.65 (chain A)
	43.49 (chain F)	22.47 (chain H)
	46.95 (chain B)	25.71 (chain B)
	47.67 (chain G)	28.41 (chain I)
GDP/Mg^{2+} /AlF_4^-/MgF_3^-	35.11/28.45/26.10 (in chain A-chain B)	13.69/15.38/11.62 (in chain A-chain B)
	34.97/28.52/25.38 (in chain F-chain G)	13.26/18.57/12.79 (in chain H-chain I)
Water	35.49	18.56
R.m.s deviations		
Bond lengths (Å)	0.0073	0.0071
Bond angles (°)	1.1851	1.1349

*Highest resolution shell is shown in parenthesis.

Table S2. Computed Structure for the transition state of the RhoA.GAP$_{R85'A}$.GTP hydrolysis. The following gives the xyz coordinates associated with the transition state structure. The number in column 1 is the atom identity, column 2 is the atomic number of the atom, columns 3, 4, and 5 give the x, y, and z coordinates in Å.

O	8	1.945222	2.845203	-0.744462
O	8	2.055915	0.567745	2.697674
O	8	-2.182352	2.650855	1.309557
C	6	-6.154686	-2.854745	4.254112
C	6	-6.171413	-3.303759	2.801922
O	8	-5.974220	-2.511553	1.883637
N	7	-6.414056	-4.611874	2.565057
C	6	-6.399536	-5.169567	1.216772
C	6	-1.965688	-0.762504	-7.205650
C	6	-2.756915	-0.994517	-5.923317
O	8	-2.786054	-0.159191	-5.029067
N	7	-3.449871	-2.162872	-5.827733
C	6	-4.246004	-2.465272	-4.650736
C	6	-3.420402	-2.426312	-3.350193
O	8	-2.234278	-2.734348	-3.321648
N	7	-4.147346	-2.065704	-2.27521
C	6	-3.572858	-1.946845	-0.944489
C	6	-2.913603	-3.246895	-0.485397
O	8	-3.460343	-4.335094	-0.632256
N	7	-1.716528	-3.083314	0.114093
C	6	-0.959094	-4.220178	0.583157
C	6	-0.318094	-5.085182	-0.520942
O	8	0.190342	-6.158922	-0.223049
N	7	-0.325632	-4.600642	-1.788861
C	6	0.313639	-5.311938	-2.882063
C	6	1.835884	-5.115764	-2.995747
O	8	2.450966	-5.743156	-3.847388
N	7	2.399517	-4.251784	-2.11997
C	6	3.833015	-4.087208	-2.010706
C	6	4.414330	-2.750306	-2.46621
O	8	5.623158	-2.641110	-2.651086
N	7	3.552845	-1.724506	-2.645164
C	6	3.984572	-0.473143	-3.234500
C	6	5.074028	0.289730	-2.470609
O	8	5.790072	1.078188	-3.074543
C	6	2.789446	0.472709	-3.389263
C	6	1.699751	-0.047094	-4.325910
C	6	0.524086	0.927034	-4.453337
C	6	-0.236390	1.169457	-3.156900
N	7	-0.830754	-0.075278	-2.622617
N	7	5.123353	0.102718	-1.133533
C	6	6.072970	0.822537	-0.325655

C	6	7.403409	0.116469	-0.103448
O	8	8.338633	0.716607	0.419616
C	6	5.516034	1.074057	1.070418
O	8	4.362612	1.930131	0.976486
N	7	7.407560	-1.187719	-0.420013
C	6	8.467744	-2.031916	0.037063
C	6	2.491624	-3.836759	7.401962
C	6	1.385562	-3.195996	6.582168
C	6	1.631861	-2.670504	5.304532
C	6	0.075314	-3.107510	7.063607
C	6	0.619535	-2.092420	4.538353
C	6	-0.945628	-2.519104	6.315839
C	6	-0.685161	-2.002536	5.04097
O	8	-1.714140	-1.451780	4.353485
C	6	-0.818600	1.773407	5.069124
C	6	-0.433552	3.192532	4.709936
O	8	-0.677583	4.166633	5.413482
N	7	0.214042	3.345676	3.518931
C	6	0.658241	4.650855	3.120507
C	6	-0.549455	5.542383	2.740736
O	8	-1.576122	5.075034	2.254402
C	6	1.556352	4.589072	1.875189
O	8	2.569107	3.610863	1.953214
N	7	-0.395893	6.882392	2.871242
C	6	-1.435550	7.825076	2.463400
C	6	4.692049	6.110842	-1.933648
C	6	3.838400	5.290619	-0.944836
O	8	2.592807	5.334434	-1.136960
O	8	4.398131	4.608148	-0.041967
C	6	0.225002	6.483233	-3.043457
C	6	-0.567826	5.413396	-2.297257
O	8	-0.150592	4.273421	-2.164803
N	7	-1.756129	5.801960	-1.722761
C	6	-2.582076	4.786322	-1.089428
C	6	-3.263219	3.872963	-2.116272
O	8	-3.705002	4.321442	-3.163116
N	7	-3.381335	2.567368	-1.740019
C	6	-3.927407	1.577643	-2.642066
C	6	-5.361192	1.137270	-2.362241
O	8	-5.871542	0.200538	-2.984241
N	7	-6.018385	1.839629	-1.420848
C	6	-7.372073	1.530625	-1.004988
C	6	-7.490848	1.563648	0.518064
C	6	-6.624689	0.528424	1.230266
C	6	-5.167774	0.932380	1.457286
N	7	-4.381868	-0.037224	1.917298
O	8	-4.786825	2.102983	1.261481
Mg	12	2.254276	1.624802	0.903057

P	15	3.392349	-2.579549	1.355419
P	15	1.276313	-1.213542	-0.116585
C	6	2.317050	-4.792835	2.312182
O	8	3.427882	-4.161103	1.668613
O	8	4.583515	-2.249447	0.525565
O	8	0.852960	-1.649317	-1.526899
O	8	3.104125	-1.809910	2.617300
O	8	2.286681	-0.074657	-0.110796
O	8	2.035017	-2.553211	0.426222
O	8	0.086408	-0.964793	0.810134
H	1	-6.798020	-1.970961	4.348875
H	1	-5.518202	-4.810314	0.663114
H	1	-6.531625	-5.229128	3.355791
H	1	-0.910031	-1.007298	-7.017088
H	1	-2.332347	-1.368281	-8.044608
H	1	-5.062797	-1.731801	-4.562221
H	1	-3.237731	-2.902828	-6.482317
H	1	-2.868963	-1.104223	-0.865433
H	1	-4.401377	-1.752994	-0.248618
H	1	-5.009233	-1.554844	-2.457854
H	1	-0.141500	-3.844254	1.213325
H	1	-1.284435	-2.157337	0.241911
H	1	0.159363	-6.391572	-2.753859
H	1	-0.734681	-3.686254	-1.971597
H	1	4.149265	-4.212779	-0.964417
H	1	4.308471	-4.859068	-2.627235
H	1	1.806245	-3.785579	-1.437112
H	1	2.389235	0.665018	-2.380852
H	1	3.179156	1.429880	-3.768965
H	1	1.335436	-1.026847	-3.973739
H	1	2.131300	-0.220459	-5.326394
H	1	0.892169	1.906098	-4.800955
H	1	-0.196205	0.567739	-5.207517
H	1	0.428456	1.565834	-2.377536
H	1	-1.038114	1.906323	-3.307571
H	1	-1.341549	0.162236	-1.740824
H	1	-0.075297	-0.791896	-2.250303
H	1	-1.458516	-0.526065	-3.297727
H	1	4.443392	-0.672155	-4.217415
H	1	2.567650	-1.835709	-2.392552
H	1	5.172637	0.130944	1.522509
H	1	4.597881	2.734233	0.464809
H	1	6.297368	1.780986	-0.817611
H	1	4.508080	-0.582558	-0.683090
H	1	9.205506	-1.400619	0.545696
H	1	6.566887	-1.609260	-0.815031
H	1	2.953246	-4.675433	6.858876
H	1	3.291658	-3.114317	7.624784

H	1	2.630127	-2.706141	4.859728
H	1	-0.158324	-3.505985	8.055375
H	1	0.851678	-1.732313	3.532184
H	1	-1.961532	-2.447899	6.708143
H	1	-1.420412	-0.954483	3.554562
H	1	-1.125314	1.748810	6.119565
H	1	0.914027	4.386610	0.999528
H	1	3.279863	3.944574	1.348075
H	1	1.185279	5.120165	3.968034
H	1	0.383462	2.553611	2.899268
H	1	-1.769727	7.567774	1.449530
H	1	0.406979	7.217493	3.386808
H	1	4.726736	5.553843	-2.881166
H	1	5.714128	6.246615	-1.560544
H	1	-0.227395	7.480160	-2.964065
H	1	-1.955425	4.201218	-0.407621
H	1	-2.238249	6.572243	-2.170448
H	1	-3.322088	0.661686	-2.621997
H	1	-3.903182	1.957454	-3.673076
H	1	-2.796864	2.201809	-0.977344
H	1	-7.224953	2.566854	0.887498
H	1	-8.546889	1.391884	0.777423
H	1	-7.033576	0.324831	2.233183
H	1	-6.644966	-0.442281	0.710728
H	1	-3.374643	0.120247	2.101825
H	1	-4.776818	-0.971507	2.017865
H	1	-7.612585	0.534853	-1.403989
H	1	-5.464750	2.476842	-0.850597
H	1	0.989573	2.855996	-0.918333
H	1	2.190600	3.813379	-0.897313
H	1	2.589192	-0.289249	2.705615
H	1	1.160993	0.217214	2.534075
H	1	-1.864850	3.414677	1.847137
H	1	1.721627	-4.062250	2.879841
H	1	1.690264	-5.294509	1.559631
H	1	-3.157329	2.488906	1.421477
H	1	8.082215	-2.788696	0.737808
H	1	8.956490	-2.555152	-0.800132
H	1	4.206290	7.077399	-2.120255
H	1	-2.303211	7.781312	3.138749
H	1	-1.014440	8.836970	2.466350
H	1	6.287180	1.544263	1.693112
H	1	2.101523	-4.219649	8.356029
H	1	-6.485042	-3.632995	4.954090
H	1	-5.127862	-2.550165	4.506151
H	1	2.719353	-5.538550	3.011400
H	1	-6.358425	-6.262040	1.291484
H	1	-7.305639	-4.877499	0.664778

H	1	-4.667032	-3.471893	-4.762242
H	1	-1.588587	-4.902133	1.169789
H	1	-8.080059	2.249996	-1.450588
H	1	-2.031270	0.303968	-7.448315
H	1	-3.361089	5.279614	-0.491606
H	1	2.001927	5.583689	1.719170
H	1	0.303995	6.185363	-4.097519
H	1	1.231503	6.461943	-2.597794
H	1	0.023285	1.086247	4.898385
H	1	-1.639875	1.436529	4.421325
H	1	-0.140110	-4.990905	-3.827945
P	15	-1.105715	0.992648	1.066216
O	8	-1.603040	0.356610	2.332499
O	8	0.193038	1.765239	1.072226
O	8	-1.819710	0.729867	-0.233760

Table S3 Calculated ^{17}O Shielding Tensors for the TS Model

Shielding tensors for O^{1G}, O^{2G}, and O^{3G}, which were calculated using methods described previously.[16]

O^{1G}: 150.1 ppm
O^{2G}: 132.1 ppm
O^{3G}: 134.3 ppm

Figure S1. Simulated annealing omit maps for (A) GDP-MgF_3^--Wat and (B) GDP-AlF_4^--Wat in the complexes of RhoA/$GAP_{Arg85'Ala}$ are shown (green mesh). (C) Overlay of PDB: **5m6x** and PDB: **5m7o**.

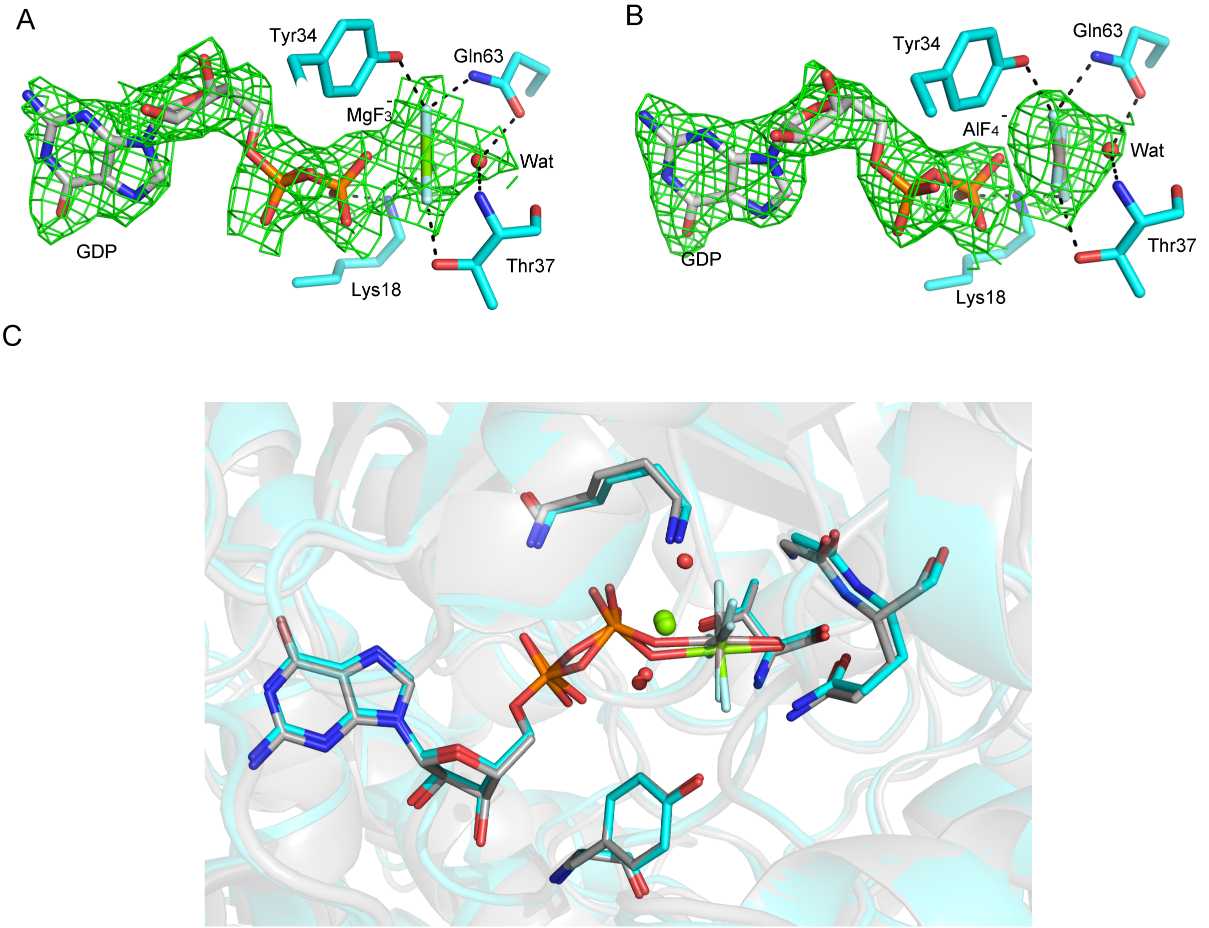

Figure S2. ^{19}F NMR spectra for (A) RhoA/GAP$_{Arg85'Ala}$-GDP-AlF_4^- TSA complex, (B) RhoA/GAP$_{Arg85'Ala}$-GDP-MgF_3^- TSA complex, (C) WT RhoA/GAP-GDP-AlF_4^- TSA complex, and (D) WT RhoA/GAP-GDP-MgF_3^- TSA complex. The SIIS values of AlF_4^- TSA complexes for both Arg85´Ala (E) and WT (F) are shown by overlaying the spectra of the complexes in 100% H_2O (in red) and 100% D_2O (in blue). Presaturation was applied on the free fluoride resonance (at around -119 ppm) for both AlF_4^- in (A and E) and MgF_3^- in (B) to eliminate the broad unbound MgF^+ peak (at -155 ppm) and AlF_x peak (at -156 ppm), for the clarity of the bound MgF_3^- and AlF_4^- signals.

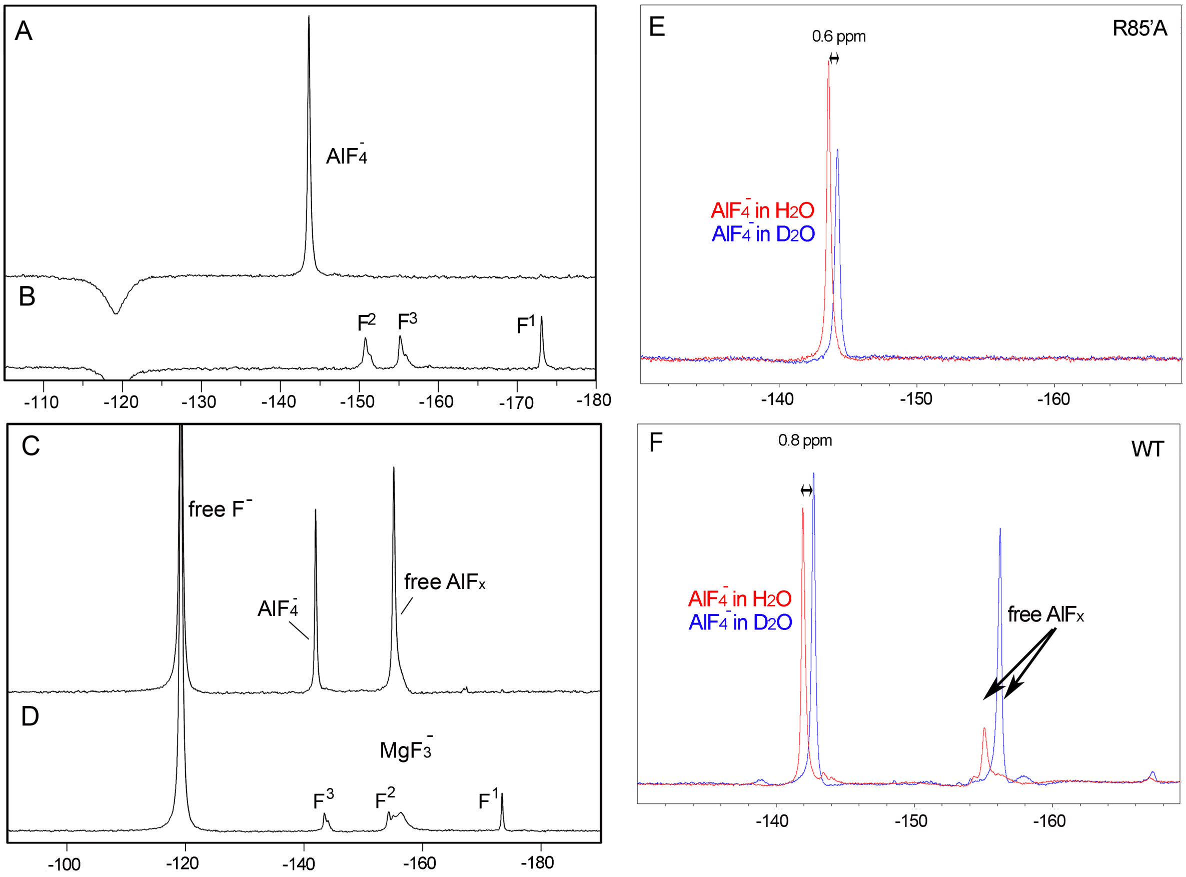

Figure S3. DFT back-calculated MgF_3^- structure (in magenta) for the catalytic core of RhoA/$GAP_{Arg85'Ala}$-GDP-MgF_3^- containing 95 heavy atoms (190 total atoms) is overlaid on **5m6x** (in cyan). The two structures are overlaid by aligning 10 terminal methyl groups on the corresponding carbons in **5m6x** (rmsd 0.12 Å for alignment of heavy atoms). Guanosine (silver) is added from PDB: **5m6x** for orientation but only C5' is included in the calculation. (Color: carbon, cyan/magenta; nitrogen, blue; oxygen, red; magnesium, green; fluorine, light blue).

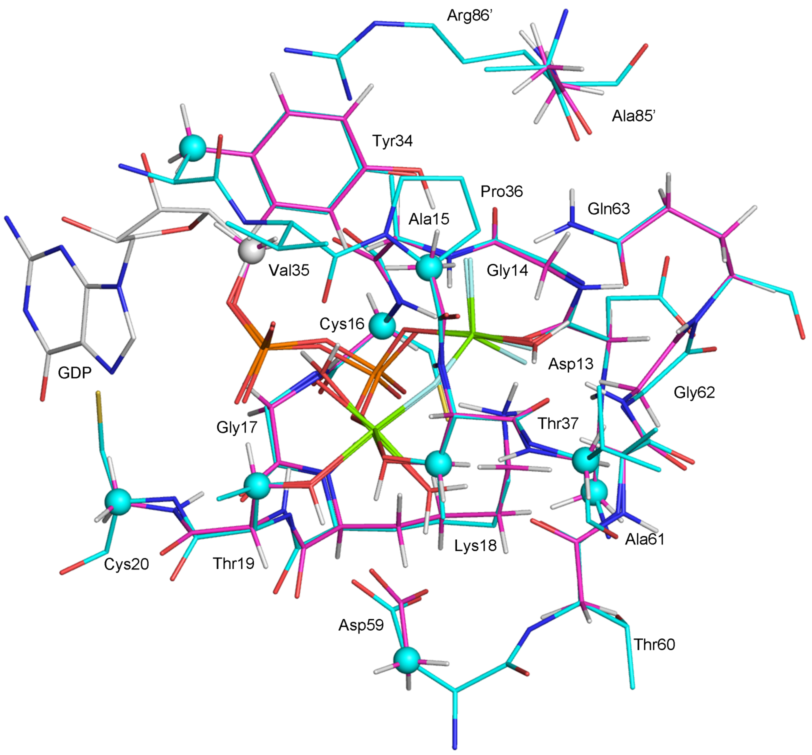

Figure S4. (A) DFT computed structure for TS of the catalytic core of RhoA/GAP$_{R85'A}$/GTP/Wat showing 20 participating residues, (silver) and 20 H-bonds (red dashes) with Tyr34 (gray) highlighted, and 10 boundary methyl carbons (cyan spheres), containing 95 heavy atoms (190 total atoms). (B) The two structures are overlaid by aligning 10 terminal methyl groups on the corresponding carbons in **5m6x** (rmsd 0.12 Å). Guanosine (cyan) is added from PDB: **5m6x** for orientation but only C5' is included in the calculation. (Color: carbon, teal green; nitrogen, blue; oxygen, red; magnesium, green; phosphorus, orange; Tyr34, black; guanosine, gray).

A

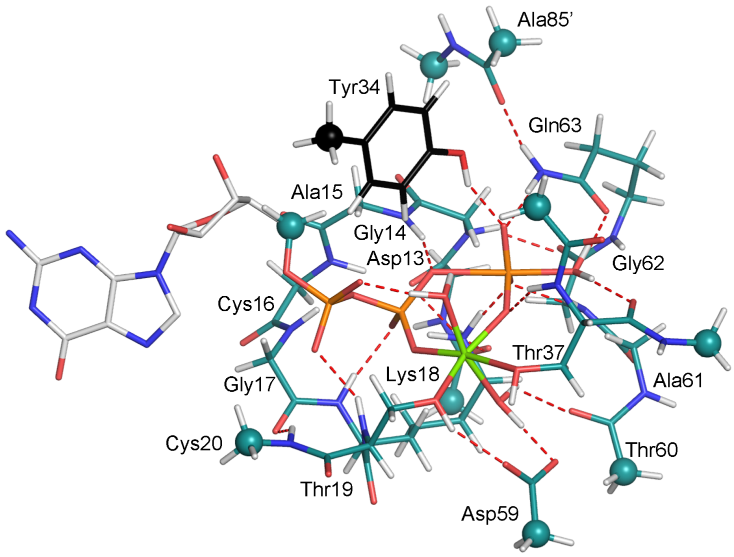

B

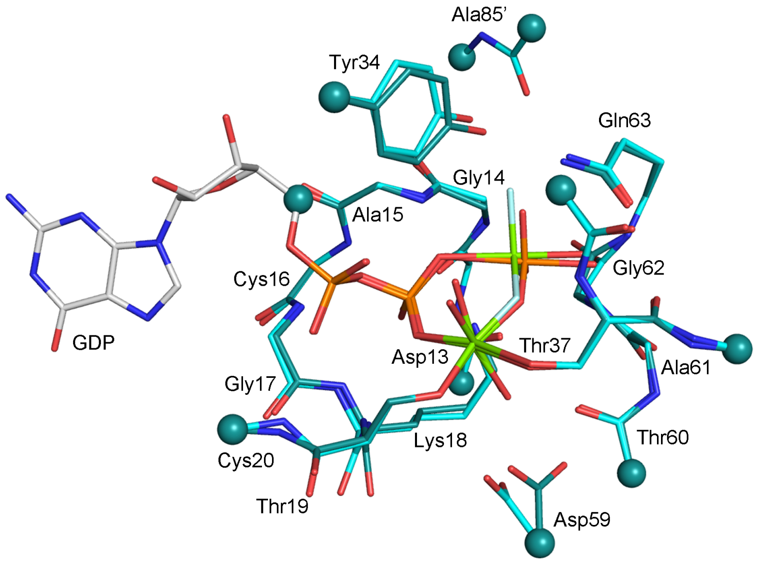

Figure S5. Maps of computed surface electron density (CD for mutant RhoA.GAP$_{R85'A}$.GTP hydrolysis transition state at four surface electron densities, contoured for (A) at 0.150 e$^-$ a_0^{-3} (1.012 e$^-$ Å^{-3}), for (B) at 0.050 e$^-$ a_0^{-3} (0.337 e$^-$ Å^{-3}), for (C) at 0.040 e$^-$ a_0^{-3} (0.270 e$^-$ Å^{-3}), and for (D) at 0.030 e$^-$ a_0^{-3} (0.202 e$^-$ Å^{-3}; the symbol a_0 designates Bohr radius). The reaction coordinate is vertical with O^{w3} top, P^G center, and O^{3B} lower. The perspective is from the rear of that in Fig. 4 with O^{3B}, P^G, O^{w3}, Mg_{cat}, OThr37 and OGln63 in the 2D plane which places Tyr34 at the rear. At the highest surface electron density (A), pairs of covalently bonded atoms are linked by intact surface electron density; there is no surface connection from P^G to O^{3w} nor to O^{3B}; yet there is intact surface electron density from O^{w3} to its two hydrogens. At intermediate surface electron density (B), there is bonding from P^G to O^{w3} but not to O^{3B}. Progressive lowering of the surface electron density (B, C & D) shows partial bonding to O^{3B} as well as good H-bonding from the two hydrogens of Wat3 to the carbonyl oxygens of Gln63 (top right) more developed than that to Thr37 (top left). (Atom coloring: hydrogen, white; carbon, gray; nitrogen, blue; oxygen, red; phosphorus, orange; magnesium, lime green, the maps are "slabbed" from the front to remove electron density mesh and frontal atoms for clarity). CD: (A) 0.150 e$^-$ a_0^{-3} (1.012 e$^-$ Å^{-3}); (B) 0.050 e$^-$ a_0^{-3} (0.337 e$^-$ Å^{-3}); (C) 0.040 e$^-$ a_0^{-3} (0.270 e$^-$ Å^{-3}); (D) 0.030 e$^-$ a_0^{-3} (0.202 e$^-$ Å^{-3}).

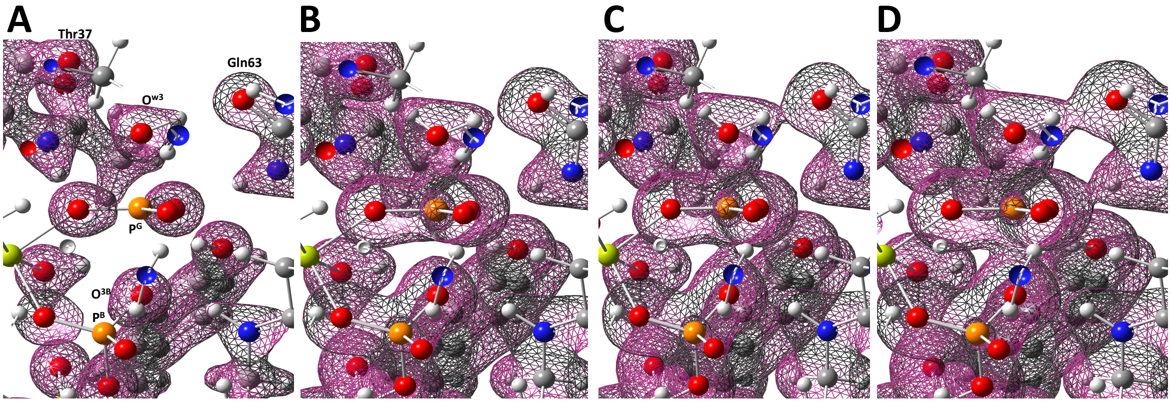

Supplement: Supplementary file 1 — Supplementary [file ANIE-56-9732-s001.pdf]
